# Synthesis of High Surface Area—Group 13—Metal Oxides via Atomic Layer Deposition on Mesoporous Silica

**DOI:** 10.3390/nano12091458

**Published:** 2022-04-25

**Authors:** Robert Baumgarten, Piyush Ingale, Kristian Knemeyer, Raoul Naumann d’Alnoncourt, Matthias Driess, Frank Rosowski

**Affiliations:** 1 BasCat—UniCat BASF JointLab, Technische Universität Berlin, Hardenberstraße 36, 10623 Berlin, Germany; r.baumgarten@bascat.tu-berlin.de (R.B.); p.ingale@bascat.tu-berlin.de (P.I.); k.knemeyer@bascat.tu-berlin.de (K.K.); matthias.driess@tu-berlin.de (M.D.); frank.rosowski@basf.com (F.R.); 2 Institut für Chemie: Metallorganik und Anorganische Materialien, Technische Universität Berlin, Straße des 17. Juni 135, 10623 Berlin, Germany; 3 Process Research and Chemical Engineering, BASF SE, Carl-Bosch-Straße 38, 67056 Ludwigshafen, Germany

**Keywords:** atomic layer deposition, thermogravimetry, metal oxides, Ga_2_O_3_, In_2_O_3_, trimethylgallium, trimethylindium, high surface area, mesoporous silica

## Abstract

The atomic layer deposition of gallium and indium oxide was investigated on mesoporous silica powder and compared to the related aluminum oxide process. The respective oxide (GaO_x_, InO_x_) was deposited using sequential dosing of trimethylgallium or trimethylindium and water at 150 °C. In-situ thermogravimetry provided direct insight into the growth rates and deposition behavior. The highly amorphous and well-dispersed nature of the oxides was shown by XRD and STEM EDX-mappings. N_2_ sorption analysis revealed that both ALD processes resulted in high specific surface areas while maintaining the pore structure. The stoichiometry of GaO_x_ and InO_x_ was suggested by thermogravimetry and confirmed by XPS. FTIR and solid-state NMR were conducted to investigate the ligand deposition behavior and thermogravimetric data helped estimate the layer thicknesses. Finally, this study provides a deeper understanding of ALD on powder substrates and enables the precise synthesis of high surface area metal oxides for catalytic applications.

## 1. Introduction

Group 13 metal oxides (e.g., Al_2_O_3_, Ga_2_O_3_, and In_2_O_3_) possess key properties for a broad range of applications such as semiconductors, optoelectronics, and catalysts. Aluminum oxide is used as an insulator in gate transistors [1], as inert fillers [2], and as ceramics due to its firmness [3]. Gallium oxide can be applied as oxygen-gas sensors [4], as surface passivation of solar cells [5], and in electroluminescent devices [6]. Because of its high optical transparency and electric properties, indium oxide is used in numerous optoelectronic applications such as photovoltaics [7], light-emitting diodes [8], and modern displays [9].

In addition to electronic applications, group 13 metal oxides are crucial components of heterogeneous catalysts. Al_2_O_3_ acts as a typical catalyst support, for example in Pt-Sn/Al_2_O_3_ which is employed industrially for the dehydrogenation of propane [10]. Ga_2_O_3_ has been studied for the dehydrogenation of light alkanes such as propane. Moreover, In_2_O_3_-based catalysts have received tremendous attention due to their ability to convert CO_2_-rich syngas into methanol [11,12]. Especially in heterogeneous catalysis, most of the reactions take place at active sites on the material’s surface. Therefore, a high surface area and homogeneous dispersion of deposited interfaces (e.g., metal oxides) are vital for enhanced activity [13].

The native bulk oxides of gallium and indium exhibit specific surface areas below 120 m^2^/g [12,14,15]. In order to increase the surface areas for catalytic applications, the oxides can be deposited on a carrier material such as porous silica, alumina, and carbon with up to 600 m^2^/g [16,17]. One well-established tool for the deposition of uniform, nanoscale films is atomic layer deposition (ALD). This technique follows sequential reactions of a gaseous precursor and a reactant with the terminal groups of a material’s surface, growing one sub-monolayer per cycle (ca. 1 Å) [18]. One of the most commonly studied materials grown by ALD is Al_2_O_3_, using trimethylaluminum (TMA) and water as a precursor-reactant combination [19]. Alumina can be deposited on substrates with various topographies such as flat silicon wafers for passivation [20], electrodes for enhanced cyclability [21], and even polymers [22].

Since ALD is applicable to materials with different topographies, it progressively gained recognition in heterogeneous catalysis [23,24]. It was also investigated as a synthesis tool for the precise deposition of active metals [25,26] or metal oxides [27,28,29]. For instance, alumina overcoating on a Pt/Al_2_O_3_ catalyst was shown to prevent the sintering of Pt during propane dehydrogenation (PDH) [30]. Additionally, an alucone layer on Ni/SiO_2_ prevented unwanted carbon formation under dry reforming conditions [31]. Further, ZnO ALD was applied to synthesize PDH-active Pt_1_Zn_1_ nano-alloys [32]. Thereby, SiO_2_ was used as a carrier material for the ZnO ALD layer to increase the specific surface area up to 400 m^2^/g. Although AlO_x_ ALD is widely applied in catalyst research, synthesis strategies employing ALD of the other group 13 oxides (e.g., GaO_x_ and InO_x_) are seldom investigated. Yet, there are some examples such as the usage of GaO_x_ ALD to introduce acid sites on zeolites [28] or the application of InO_x_ ALD to grow an In_2_O_3_ layer over Pt/Al_2_O_3_ as an efficient PDH catalyst [29].

To date, published studies on the deposition behavior of GaO_x_ or InO_x_ ALD on powder substrates are limited, especially with regard to higher surface area and porous structure [33]. On flat substrates, however, different precursor-reactant combinations were studied for the deposition of GaO_x_ such as GaMe_3_/O_2_-plasma [34,35,36,37], GaEt_3_/O_2_-plasma [38,39], Ga(^i^OPr)_3_/H_2_O [40], Ga(CpMe_5_)/ O_2_ + H_2_O [41], and [Ga(NMe_2_)_3_]_2_/O_2_-plasma [42]. Yet, all of them aimed for coatings of Si-wafer, fused silica, or SiO_2_ terminated Si, focusing for example on electronic applications such as thin-film transistors [43]. The same accounts for InO_x_ ALD investigations on flat substrates which comprise the usage of numerous combinations such as InCl_3_/H_2_O [44], InMe_3_/(H_2_O or O_2_-plasma) [45,46,47,48], InEt_3_/O_2_-plasma [39], InCp/O_2_ + H_2_O [49,50], and others [46,51,52,53,54,55,56,57,58].

The consensus of the studies collected above is that water as an oxygen source, especially in combination with GaMe_3_, leads to low growth rates due to the insufficient removal of methyl ligands [35]. Similar conclusions were made for the combination of InMe_3_ and water [47]. Nevertheless, Kim et al. [45] found that a longer Langmuir exposure of H_2_O (ca. 2 Torr·s) enabled the complete exchange of methyl groups, yielding In_2_O_3_ with linear growth per cycle (gpc). These findings can be rationalized by high activation barriers to remove the methyl group through water (E_a_(Ga-CH_3_) = 151.0 and (In-CH_3_) = 169.8 kJ/mol), calculated by Shong et al. [59]. Insufficient ligand removal can be overcome by the usage of reactants with higher oxidation potential such as O_2_-plasma [47]. However, plasma has the drawback of swift recombination on larger steel set-ups [60,61] and might lead to unwanted changes in the surface morphology of the substrate [62]. In addition to ALD, metal organic chemical vapor deposition (MOCVD) was used to synthesize defined layers of indium oxides. For example, Kakanakova-Georgieva et al. managed to stabilize two-dimensional (2D) layers of InO between graphene and Si/C *via* MOCVD of InMe_3_ [63]. Hereby, DFT calculations were applied to investigate bonding and structure particularities, revealing a sequence of O-In-In-O for the 2D InO quadruple layer [64]. However, on amorphous silica, the formation of highly ordered oxides is unlikely as the surface structure is far more complex than ordered Si/C.

In order to use the full potential of ALD for the modification of porous substrates, deeper knowledge about the deposition mechanisms on powders is essential. Additionally, ALD on powders demands different process parameters which are less relevant for the coating of flat substrates [65,66]. For instance, diffusion limitations in the pores and high surface areas (100–500 m^2^/g) require different reactor geometries and longer dosing times [66,67]. Fixed- or fluidized-bed reactors were shown to be convenient, however, they cannot accommodate spectroscopic ellipsometry or a quartz crystal microbalance (QCM) for in-situ monitoring. Therefore, ALD processes on powders such as AlO_x_/SiO_2_ [68], ZnO/SiO_2_ [67], and PO_x_/V_2_O_5_ [69] were studied using a magnetic suspension balance for in-situ thermogravimetric analysis [70]. In the current study, we progressed with detailed investigations of the ALD growth behavior of gallium and indium oxides on mesoporous silica powder as a model system. The respective oxide was deposited with up to three cycles by the sequential dosing of trimethylgallium (TMG)/H_2_O and trimethylindium (TMI)/H_2_O at 150 °C.

## 2. Materials and Methods

### 2.1. Materials

Silica powder (SiO_2_ amorphous, ≥99%, high-purity grade (Davisil Grade 636), average pore size 60 Å, particle size 250–500 µm, specific surface area 505 m^2^/g, Sigma-Aldrich, St. Louis, MO, UAS) was used as a substrate for atomic layer deposition. Trimethylaluminium (Al(CH_3_)_3_, TMA, elec. grade (99.999%—Al)), Trimethylgallium (Ga(CH_3_)_3_, TMG, elec. grade (99.999%—Ga)), and Trimethylindium (In(CH_3_)_3_, TMI, elec. grade (99.999%—In)) (Strem Chemicals Europe, Bischheim, France) were employed as atomic layer deposition precursors. Water (H_2_O, CHROMASOLV^®^, for HPLC, Riedel-de Haën/ Honeywell Specialty Chemicals Seelze GmbH, Seelze, Germany) served as a reactant and was used without further purification. High purity argon (Ar, 99.999%) was used as a carrier and purging gas.

### 2.2. Atomic Layer Deposition of GaO_x_ and InO_x_ on SiO_2_

Initially, the deposition behavior was examined in a magnetic suspension balance (MSB) for in-situ monitoring of mass changes (marked with MSB or in-situ). Afterward, the developed processes were scaled up in a quartz tube fixed bed reactor, producing up to 20 mL of ALD-modified material for ex-situ analysis. Both self-build setups possess fixed bed geometry operating at atmospheric pressure with top-to-bottom flow, as further described elsewhere [70]. In the MSB, GaO_x_, and InO_x_ ALD was carried out under a constant flow of 50 mL/min containing precursor or reactant diluted in argon. For each cycle, reactants were dosed until no further mass change was detected to ensure saturation. The same procedure accounted for intermediate purging steps to ensure the removal of gaseous precursors. For the larger fixed bed reactor (FB), a continuous flow of 100 mL/min was applied and saturation of the precursor was determined by an online quadrupole mass spectrometer (Pfeiffer Vacuum, Asslar, Germany). The point of saturation is reached once a constant ion current of unreacted precursor ions is measured in the MS shortly after the signal breakthrough (TMG: 69 *m*/*z* for Ga* and TMI: 115 *m*/*z* for In*), similar to previously described [67]. The precursor chamber of TMG was kept at RT and TMI at 80 °C while the reactors were maintained at 150 °C. For both oxides, three cycles were performed employing an ALD-sequence (cycle) of TMX/Ar-purge/H_2_O/Ar-purge on dried silica powder.

### 2.3. Characterization of the Materials

Nitrogen physisorption measurements were performed at liquid N_2_ temperature (77 K) using a Quadrasorb SI (Quantachrome GmbH & Co. KG, Odelzhausen, Germany). Prior to measurements, the samples were degassed at 150 °C for 2 h. The specific surface areas were determined applying the B.E.T. method (Brunauer–Emmett–Teller) and the corresponding pore size distribution was calculated from the desorption branches using the B.J.H. method (Barrett–Joyner–Halenda). Powder X-ray diffraction (XRD) patterns were acquired with an X‘PERT Pro (PANalytical, Malvern, UK) equipped with a scintillation detector, using Cu K*α*1 radiation (*λ* = 0.154 nm). Inductively coupled plasma optical emission spectrometry (ICP-OES) was employed to determine In and Ga contents and measured on a Varian 720-ES (Varian Inc., Palo Alto, CA, USA). Solutions from the powder were prepared via acidic leaching. Respective metals were detached from silica in a sealed container using saturated hydrochloric acid (35%) at 120 °C. The spectroscope was three-point calibrated with a commercially available, diluted standard for In and Ga. Mass fractions of carbon, hydrogen, nitrogen, and sulfur were determined by combustion analysis (CHN), executed on a EuroEA Elemental Analyzer (HEKAtech GmbH, Wegberg, Germany). FT-IR spectroscopy was measured in transmission (4000–400 cm^−1^) on a Bruker ALPHA FT-IR spectrometer inside a glove box. Samples were diluted with KBr, ground in a mortar, and pressed into pellets. Prior to preparation, samples were dried at 130 °C for 3 h and transferred into the glovebox. Spectra were collected as data point tables by the usage of OPUS (Bruker, Billerica, MA, USA). Solid-state (SS) nuclear magnetic resonance (NMR) spectra were recorded with a Bruker Avance 400 MHz spectrometer operating at 100.56 MHz for ^13^C and 79.44 MHz for ^29^Si. High-power decoupled (HPDEC) ^13^C and ^29^Si cross-polarization magic angle spinning (CP/MAS) NMR experiments were carried out at a MAS rate of 10 kHz, contact time of 2.0 ms, and a recycle delay of 2 s, using a 4 mm MAS HX double-resonance probe. Spectra are referenced to those of external tetramethylsilane (TMS) at 0 ppm for ^13^C and ^29^Si, using adamantane and tetrakis(trimethylsilyl)silane (TKS) as secondary references, respectively. X-ray photoelectron spectroscopy (XPS) was carried out on a K-Alpha™ + X-ray Photoelectron Spectrometer System (Thermo Fisher Scientific, Waltham, MA, USA), equipped with a Hemispheric 180° dual-focus analyzer connected to a 128-channel detector. The X-ray monochromator applies micro-focused Al−Kα radiation. The as-prepared samples were loaded directly on the sample holder for measurement. Data were collected with an X-ray spot size of 200 μm, 20 scans for the survey, and 50 scans for regions. Binding energy surveys were calibrated according to the C1s orbital fixed at 284.8 eV. Scanning transmission electron microscopy (STEM) was performed on an FEI Talos F200X (Thermo Fisher Scientific, Waltham, MA, USA) with an XFEG field emission gun and acceleration voltage of 200 kV. Energy-dispersive X-ray (EDX) mappings were recorded with a SuperX system of four SDD EDX detectors (Analysis software: Velox 2.9.0 by Thermo Fisher Scientific, Waltham MA, USA). The surface density of OH groups was determined via a Grignard titration method described in detail elsewhere [71]. A j-young NMR tube was loaded with ca. 20 mg of SiO_2_ (dried at 150 °C), ferrocene, and self-synthesized Mg(CH_2_Ph)_2_·2(THF) as Grignard reagent (mass-ratio ca. 3:1:5) inside a glovebox. The solid mixture was suspended in benzen-d6 and the NMR tube was sealed and shaken to let the reagents react with the OH-groups of SiO_2_. ^1^H NMR spectra were measured on a Bruker Avance II 200 MHz spectrometer (Bruker BioSpin MRI GmbH, Ettlingen, Germany). The total number of OH-sites was determined by calculating the number of moles of toluene produced (based on its methyl group peak integral at 2.1 ppm) using ferrocene as an internal standard.

## 3. Results

### 3.1. In-Situ Thermogravimetric Analysis

**Figure 1:**
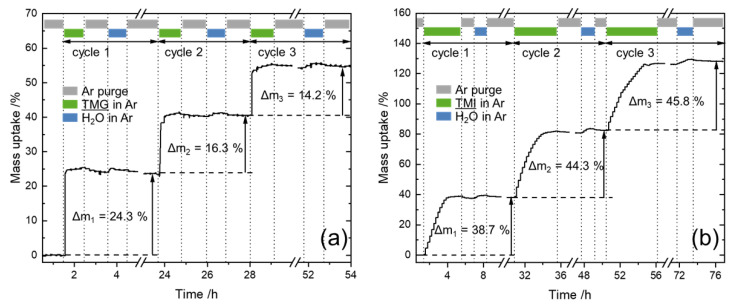
In-situ gravimetric monitoring of (**a**) GaO_x_ ALD and (**b**) InO_x_ ALD on SiO_2_ powder at 150 °C using the ALD processes of TMG/H_2_O and TMI/H_2_O, respectively. Mass-uptake = ∆m/m_0_.

Deposition of GaO_x_ and InO_x_ was carried out on mesoporous SiO_2_ using the ALD processes of TMG/H_2_O and TMI/H_2_O at 150 °C. The mass change during ALD was monitored using an in-situ magnetic suspension balance (MSB, Figure 1). Both ALD processes showed self-limitation for all first half-cycles when the precursor is dosed as well as during the ligand removal steps in the second half-cycles, representing ALD growth behavior. In the case of GaO_x_ ALD, self-limitation of precursor chemisorption was reached within minutes, as for InO_x_, the first half-cycles extended over two hours. This can be rationalized by the three times higher vapor pressure of the gallium precursor under our conditions (TMG_300K_ = 327 mbar [72] and TMI_353K_ = 107 mbar [73]). Subsequently, the slight increase in mass during the second half-cycles relates to the exchange of the methyl group (15 g/mol) by the heavier OH-groups (17 g/mol) introduced by water.

In the following, the trend of growth is discussed based on the in-situ mass-uptake, defined as the mass deposited by ALD divided by the initial mass of the support (Table 1). In the first full cycle, the GaO_x_ ALD led to a mass-uptake of 24.3 wt% while the uptake declined within the second and third cycles to 16.3 and 14.2 wt%. This indicates either a substrate enhanced growth or incomplete ligand removal in the second half-cycles, as further discussed in the following section.

Interestingly, Elam et al. observed a declining ALD growth of GaO_x_ due to insufficient removal of methyl ligands using TMG/water above 200 °C [35]. Our in-situ gravimetric studies also indicated lower GaO_x_ uptakes (−33%) after the first ALD cycles. However, the use of H_2_O as a reactant did not hinder distinct growth during subsequent cycles. Moreover, a fourth cycle was conducted (Figure S1), resulting in uptakes of 13.4 wt% GaO_x_ being of the same order of magnitude as the third cycle (14.2 wt%). Therefore, incomplete ligand removal does not necessarily translate to full inhibition of further growth.

In contrast, the InO_x_ ALD led to a mass-uptake of 38.7 wt% in the first cycle and increased to 44.3 and 45.8 wt% in the following cycles. Increased uptake at higher cycle numbers hints at higher reactivity between the precursor and deposited oxides or a higher abundance of OH-groups compared to SiO_2_. Elam et al. demonstrated poor nucleation employing TMI/H_2_O in a quartz crystal microbalance and therefore proposed using O_2_-plasma [47]. Nevertheless, our study clearly demonstrates the constant growth of InO_x_ on SiO_2_, indicating the suitability of H_2_O as a reactant. A similar observation was made by Kim et al. for the deposition of InO_x_ on a SiO_2_ terminated silicon flat substrate [45].

Under the rough assumption, that the deposited oxides have a stoichiometry of M_2_O_3_ (M = Al, Ga or In), the molar uptakes per cycle were calculated based on the thermogravimetric data (Table 1). For each oxide and ALD cycle, the deposited moles of M_2_O_3_ per gram SiO_2_ are around 1 mmol/g. This indicates similar deposition behavior for each oxide and the deposited mass is a function of the molar mass (Table S1 in Supplementary Materials).

Additionally, assuming a stoichiometry of M_2_O_3_ (M = Ga or In), the estimated metal contents were 14.5 wt% (Ga) and 23.1 wt% (In) after the first ALD cycle in the magnetic suspension balance (Table 2). The contents determined by ICP-OES were 14.6 wt% (Ga) and 21.1 wt% (In) which points to a stoichiometry of M_2_O_3_ for GaO_x_. Therefore, condensation of Ga(OH)_x_ species might already occur in the first cycle. However, the mass fraction of indium is overestimated for the first cycle, indicating that the actual deposited species has a lower mass fraction of indium than in In_2_O_3_.

Considering chemisorbed In(OH)_2_ instead delivered an estimated indium content of 21.5 wt% which matches the measured content of 21.1 wt% (In). For subsequent cycles, the estimated indium contents have a better fit to ICP-OES when In_2_O_3_ is assumed. Hence, chemisorbed In(OH)_2_ might resist condensation and do not collapse towards oxidic species in the first cycle. In subsequent cycles, the formation of In_2_O_3_ is favored during the reaction with TMI and water.

### 3.2. Effect of ALD on Surface Area and Pore Size

**Figure 2:**
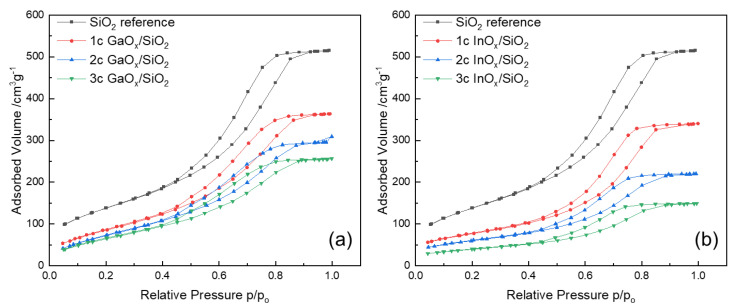
N_2_ physisorption isotherms of SiO_2_ coated with (**a**) 1–3 cycles GaO_x_ ALD and (**b**) 1–3 cycles InO_x_ ALD, using TMX (X = G or I) and water at 150 °C substrate temperature.

N_2_ physisorption measurements were conducted to analyze ALD-induced changes to the surface area and pore structure of silica. The resulting isotherms are shown in Figure 2 and the differential pore size distributions are displayed in the supplementary material. All samples led to a type IV(a) isotherm with a mixture of type H1 and H2(b) hysteresis loop, characteristic of capillary condensation in materials with larger mesopores (>4 nm).

The contribution of the H1 mode derives from equilibrium (liquid-vapor) transitions at cylindrical pores, indicated by mostly parallel adsorption and desorption branches. The addition of the H2(b) mode is associated with delayed phase transition at more complex structures, such as ink-bottle-shaped pores with a wide distribution of neck sizes [74,75]. For all samples, the overall shape of the hysteresis is not affected by ALD indicating maintained pore character and conformal coating with GaO_x_ and InO_x_ [76].

The volume of adsorbed N_2_ decreased as a function of the ALD cycle number which translates to the loss of specific surface area. The related values, calculated from the N_2_ isotherm using the BET method, are shown in Table 3. A stepwise decrease in specific surface area from 505 to 259 m^2^/g was observed within three ALD cycles of GaO_x_. At the same time, the total pore volume was approximately halved from 0.79 to 0.39 cm^3^/g. Furthermore, the InO_x_ ALD showed an even more pronounced effect as the specific surface area decreased to 142 m^2^/g and the pore volume to 0.23 cm^3^/g after three cycles.

However, the drastic decline of the specific surface area can be rationalized by the significant change in density induced by ALD. With a rising mass fraction of the deposited oxide, the sample exhibits less volume and surface area per gram. For instance, a given quantity of silica reaches 1.83 times its initial mass after two cycles of InO_x_ ALD (Table 1). Consequently, the mass-related (specific) surface area would decrease from initially 505 to 276 m^2^/g, assuming no change in the exposed surface area. The estimated value is in good agreement with the measured value of 216 m^2^/g. Similar findings were made for the AlO_x_ process on porous silica [68]. In fact, the observed changes are in a reasonable range as demonstrated in detail in the supplementary materials (Tables S2–S4 and Schemes S1–S3).

Additionally, the pore size distributions were calculated using the BJH method based on the rough assumption of having only regular and cylindrical pores (Figures S2 and S3 in Supplementary Materials). In both cases, ALD led to an even shift to smaller pore diameters with increasing cycle numbers. At the same time, the absolute desorption volume decreased, as it is also normalized to the mass of the sample. Both phenomena indicate that the pores of all diameters are decorated and accessible by the precursors. However, the calculated distribution has to be treated with reservation as the silica substrate featured an irregular and unknown system of different pore types [74,75].

### 3.3. Investigation of the Formed Phase and Its Dispersion

**Figure 3:**
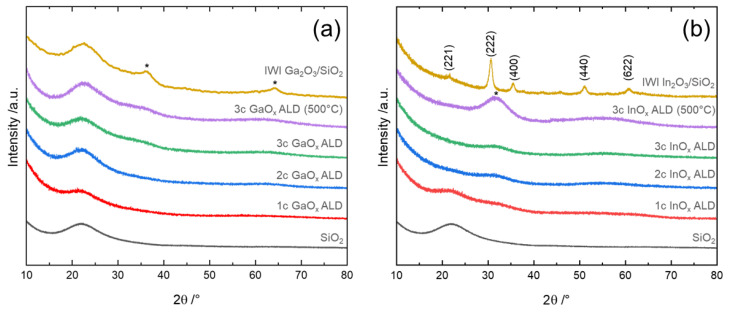
X-ray diffractograms of (**a**) 1–3 cycle GaO_x_ ALD and (**b**) InO_x_ ALD on SiO_2_ (500 °C) indicate calcination at 500 °C in 20% O_2_ for 3 h. Ga_2_O_3_(19 wt%)/SiO_2_ and In_2_O_3_(27 wt%)/SiO_2_ were synthesized via incipient wetness impregnation (IWI).

Powder X-ray diffraction (XRD) was employed to rule out the formation of crystalline agglomerates greater than the typical detection limit of around 2 nm [77,78]. The X-ray diffractograms of the as-deposited GaO_x_ show no crystalline phase after three ALD cycles, thus being XRD amorphous (Figure 3). Moreover, the broad reflection at 21.8° (2*θ*), which accounts for amorphous SiO_2_, and the diffractograms provide no defined intensity features.

X-ray amorphous GaO_x_ films were also deposited by Biyikli et al. [36] until 500 cycles of TMG/O_2_-plasma. In their case, the transition to crystalline Ga_2_O_3_ only occurred under annealing in N_2_. However, calcination of the three-cycle sample at 500 °C (20% O_2_) did not result in the formation of detectable crystallites. This underlines the stability and dispersion of the layer despite a mass-fraction of 35 wt% GaO_x_. As a reference, 19 wt% Ga_2_O_3_ was supported on silica using the incipient wetness impregnation method with gallium nitrate (Ga_2_O_3_/SiO_2_) (SI). After calcination at 500 °C, the impregnated sample exhibited broad signals characteristic of *α*- or *β*-Ga_2_O_3_ [79].

In the case of InO_x_ ALD, the x-ray diffractograms exhibited no distinct reflections, being also XRD amorphous (Figure 3). On the contrary, an additional phase centered around 31.6° (2*θ*) emerged in the diffractogram of the three-cycle sample after calcination. It lies close to the reflection of the (222) plane of cubic In_2_O_3_ typically located around 30.6°(2*θ*) [80]. It might also be an indication for the (200) facet of In(OH)_3_ lying between 31 and 32° [81]. However, the new phase might still be nanocrystalline and only the starting point for the formation of In(OH)_3_ or In_2_O_3_ crystallites.

These findings agree with the literature, as Elam et al. showed that defined reflections of the (222) plane of In_2_O_3_ only appear upon 800 cycles after annealing or at higher deposition temperatures [47]. For comparison, 27 wt% In_2_O_3_ was supported on silica via incipient wetness impregnation and calcined at 500 °C (In_2_O_3_/SiO_2_). The impregnated sample showed sharp reflections characteristic of cubic In_2_O_3_. Therefore, the impregnated In_2_O_3_ was clearly agglomerated and crystallized, while ALD provided a more dispersed InO_x_ species.

**Figure 4:**
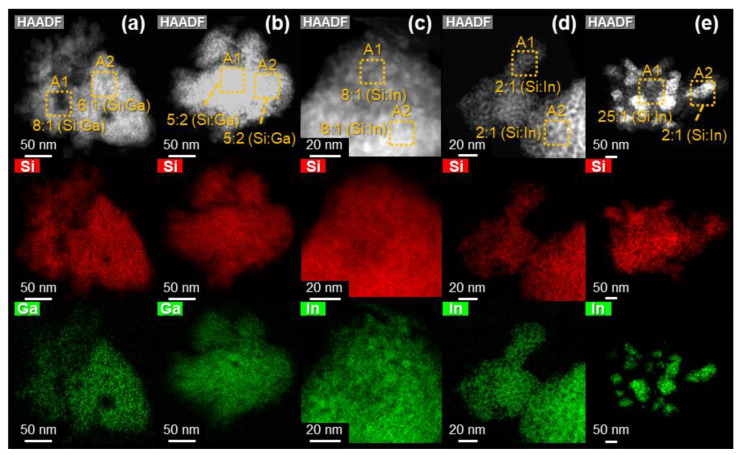
STEM-HAADF images and EDX-mappings of (**a**) one ALD cycle GaO_x_, (**b**) three-cycle GaO_x_, (**c**) one cycle InO_x_, (**d**) three-cycle InO_x_ and (**e**) impregnated In_2_O_3_ (27 wt%) on mesoporous SiO_2_. Respective energy dispersive spectra are displayed in the supplementary materials (Figures S4–S8). Atomic ratios between Si and Ga or In are indicated in the HAADF images in orange and based on the EDX-detector counts.

STEM and EDX-mapping revealed agglomeration of the impregnated In_2_O_3_ sample with particle sizes between 20 and 100 nm (Figure 4). The calculated crystal diameter based on the 35.5° (2*θ*) reflection was approximately 21 nm, applying the Debye–Scherrer equation (FWHM = 0.82° (2*θ*)). Consequently, the atomic ratios between Si and In varied significantly between 25:1 and 2:1 within the mapping. EDX-mappings of the ALD samples of InO_x_ and GaO_x_ on SiO_2_ demonstrate the opposite, without changes in morphology compared to the underlying support material. In the case of GaO_x_, the atomic ratio of Si and Ga varied between 6:1 to 8:1 in selected areas after one cycle (Figure 4).

Increased loading of the three-cycle sample led to lower Si:Ga ratios (5:2) while being contained over the whole sample which indicates high dispersion. The same was found for the InO_x_ ALD samples while the In:Si ratios were 8:1 in the one cycle and 2:1 in the three-cycle sample.

**Figure 5:**
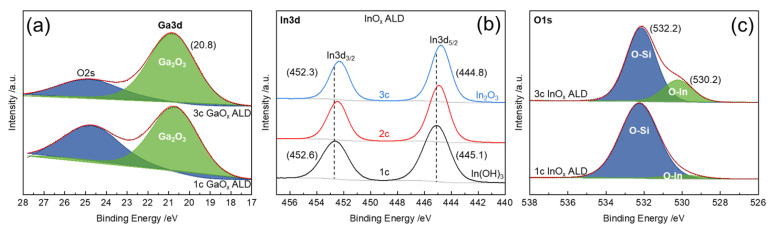
X-ray photoelectron scans of the (**a**) Ga3d, (**b**) In3d, and O1s regions of as-deposited 1–3 cycle (**a**) GaO_x_ and (**b**,**c**) InO_x_ ALD on SiO_2_. Intensities were normalized to values between 0 and 1 for comparison. Surveys were corrected to the C1s orbital peak at 284.8 eV.

X-ray photoelectron spectroscopy (XPS) was conducted to determine the oxidic species of as-deposited InO_x_ and GaO_x_ on SiO_2_. The photoemission spectra of the Ga3d region showed two overlapping peaks (Figure 5a). One can be assigned to Ga, bound with oxygen as in Ga_2_O_3_, located at 20.8 eV. The second derives from the O2s orbital, around 24.8 eV [82,83]. With increasing GaO_x_ cycle number, the intensity of the Ga_2_O_3_-related peak (Ga3d) increases in relation to the O2s peak. Decreasing contribution of the O2s signal might be the result of the increased degree of coverage of substrate oxygen (SiO_2_) by gallium oxide species. An alteration of hydroxylated Ga content was not observed (typ. around 19.6 eV) [84].

The peaks associated with the electron binding energies (BE) of the spin-orbit-coupled In3d orbitals (J = 3/2 and 5/2) are shifted to the lower BE with increasing cycle number (Figure 5b). After one ALD cycle of InO_x_, the In3d_5/2_ signal appeared at BE of 445.1 eV which can be assigned to In(OH)_3_ (445.2 eV [85]). After two cycles, the peak was located at 444.9 eV and after three cycles at 444.8 eV, while the latter is matching the binding energy as in In_2_O_3_ (444.7 eV [85]). Therefore, the deposited InO_x_ species might transition from In(OH)_x_ to In_2_O_3_ species with increasing cycle number. Additionally, increasing content of In_2_O_3_ could be observed within the O1s region (Figure 5c). The related signal was fitted into two peaks at about 532.2 eV and 530.2 eV after the third cycle. The former can be assigned to the oxygen of silica (O-Si) [86] and the second corresponds to oxygen as in In_2_O_3_ (O-In) [81,87,88]. Thereby, the ratio between Si and In_2_O_3_-related oxygen increased from 20:1 after the first cycle to 3:1 after the third cycle (see also Table S5 in Supplementary Materials).

### 3.4. Determination of Ligand Implementation

The InO_x_ ALD process led to negligible carbon contamination. CHN analysis revealed carbon contents of 0.08, 0.16, and 0.14 wt% after the first, second, and third ALD cycles. The number of moles of deposited indium equating with the moles of incorporated precursors can be determined from the indium content. The ratio between moles of methyl groups of used precursor and moles of carbon provides information about the number of methyl groups still being attached after the dosing of water. As a result, approximately 1 out of 105 methyl groups of the deposited precursor remained, on average, after each cycle.

**Figure 6:**
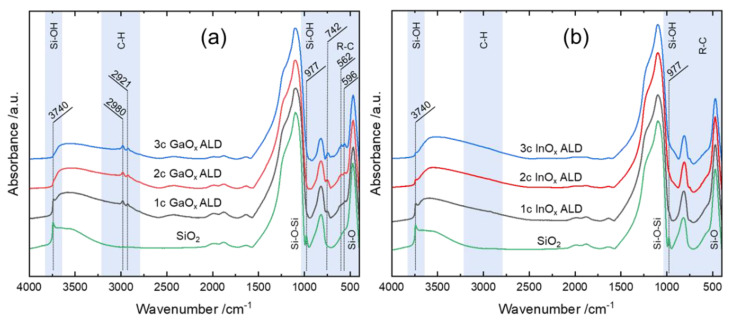
FTIR spectra (4000–400 cm^−1^) of 1-3 cycle (**a**) GaO_x_ and (**b**) InO_x_ ALD on silica using TMX (X = G, I) and water at 150 °C.

**Figure 7:**
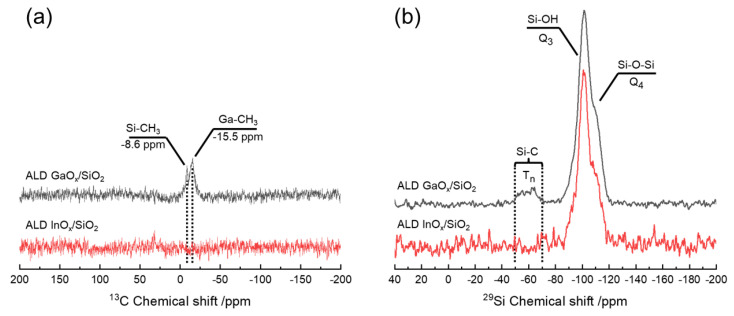
(**a**) (HPDEC) ^13^C and (**b**) ^29^Si CP/MAS SS-NMR spectra of as-deposited one ALD cycle GaO_x_ and InO_x_ on SiO_2_ (ppm rel. to TMS).

Ultimately, the question arises where the carbon species is deposited and of which nature it is. In addition to the typical absorption bands of SiO_2_, the FTIR spectra of InO_x_/SiO_2_ showed no features in the C-H stretching regions (Figure 6). The silanol band around 3740 cm^−1^ and 977 cm^−1^ decreased in intensity with higher cycle numbers as TMI chemisorbed on Si-OH. Still, the silanol-related bands persisted as weak shoulders after the third cycle. This indicates unreacted Si-OH groups that might be sterically blocked or in the bulk. For further evaluation of carbon species solid-state (SS), NMR analysis is necessary. Yet, the amount of carbon was not sufficient to induce changes in the silicon environment visible in the T_n_ zone (−50 to 80 ppm) of the ^29^Si SS-NMR spectrum or signals in the ^13^C SS-NMR spectra (Figure 7).

Conversely, the GaO_x_ ALD process provided carbon contents of 1.84 (1c), 2.56 (2c), and 2.70 wt% (3c) measured via CHN analysis. The amount of carbon after the first cycle was therefore in the same order of magnitude as for our AlO_x_/SiO_2_ process (1.52, 1.59, and 1.33 wt% C [68]). Since the carbon content did not further increase after the second cycle of AlO_x_, the favored methylation of underlying SiO_2_ might be the reason for carbon contamination. Conducting the same calculation as for InO_x_ suggests that 1 out of 8 methyl groups persist during the re-hydroxylation of the first cycle and 1 out of 19 persist within three cycles of AlO_x_. In the case of GaO_x_, the carbon content increased further for subsequent cycles indicating different carbon deposition behavior. Hereby, one out of five methyl groups remained on the substrate after the first cycle and one out of six within three cycles. A constant proportion of un-removed carbon in each cycle hints at methylated gallium as the contact of TMG to underlying silica becomes less likely with a higher cycle number.

The FTIR spectra of GaO_x_/SiO_2_ (Figure 6) held two sharp absorption bands in the C–H stretching region at 2980 and 2921 cm^−1^ which can be attributed to [*_νas_*C-H] and [*_νs_*C-H] of methyl species [89,90,91]. Hereby, Si-CH_3_ is formed through the dissociative chemisorption of TMG on Si-O-Si, and methoxy species were not found [92,93]. The band at 2980 cm^−1^ is essentially shifted to a higher wavenumber and therefore attributed to methylated gallium as reported by Ring et al. [93]. Additionally, new features emerge in the fingerprint region at 742, 596, and 562 cm^−1^. The band at 742 cm^−1^ is assigned to the CH_3_ rocking mode, while 596 and 562 cm^−1^ are related to [*_νas_*Ga-C_2_] and [*_νs_*Ga-C_2_]. This underlines the formation of resilient, di-methylated gallium [81]. These findings are in line with the results of Elam et al. as they also detected CH_3_ species via FTIR which were not fully removed upon exposure to water [35]. At the same time, the bands corresponding to silanol at around 3740 and 977 cm^−1^ disappeared after the third cycle, indicating nearly full coverage of Si–OH between the second and third cycles.

In the present case, methylation was also observed in the ^13^C SS-NMR spectra as broad signals with maxima positioned around −8.6 and −15.5 ppm chemical shift (Figure 7). The peak closest to 0 ppm can be assigned to mono-methylated silicon (O_3_–Si–CH_3_) and the signal more up-field might derive from methylated gallium (O-Ga-CH_3_ or O-Ga-(CH_3_)_2_) [94,95]. The formation of methoxy species or longer alkyl chains can be ruled out as they typically appear down-field at around 50 or 22 ppm [89,96,97]. Methylation of gallium indeed explains inhibition of uptake as full recreation of hydroxyl termination is impossible. Nevertheless, OH-groups and bridging oxygens were sufficiently available on the outer layer of GaO_x_ which resulted in distinct growth in all cycles.

The presence of alkyl species in the vicinity of Si was also observed in the ^29^Si SS-NMR spectrum of GaO_x_/SiO_2_ (Figure 7). The broad signal reaching from −50 to −70 ppm agrees with the classic T_n_ zone of alkylated SiO_2_ [98]. Within the T_n_ zone, the maxima found between −55 and −59 ppm can be assigned to T_2_ (HO-Si-CH_3_) being attached to two bridging oxygen of the silica bulk. The maxima in the T_3_ region, located between −62 and −65 ppm, are related to Si-CH_3_ being connected to three bridging oxygen [94,99,100,101].

This essentially proves the methylation of silicon during the GaO_x_ ALD process. Methylation of silicon mostly originates from the dissociation of precursors on oxygen-bridged silicon. Therefore, the TMG precursor might favor the dissociation reaction more than TMI. Finally, the distorted peak positioned between −85 and −120 ppm is clearly assignable to silanol groups and bridged silicon of Q_3+4_ (O_3_-Si-OH, O_4_-Si) [100,102].

### 3.5. Decryption of the Growth Mechanism

Combining in-situ thermogravimetric data with ICP-OES facilitates the determination of a tendency of the ligand exchange mechanism during the first cycle of the GaO_x_ and InO_x_ ALD. The carbon content is neglected for convenience and multiple dissociation steps are excluded as repeated dissociation does not lead to further change in mass.

**Figure 8:**
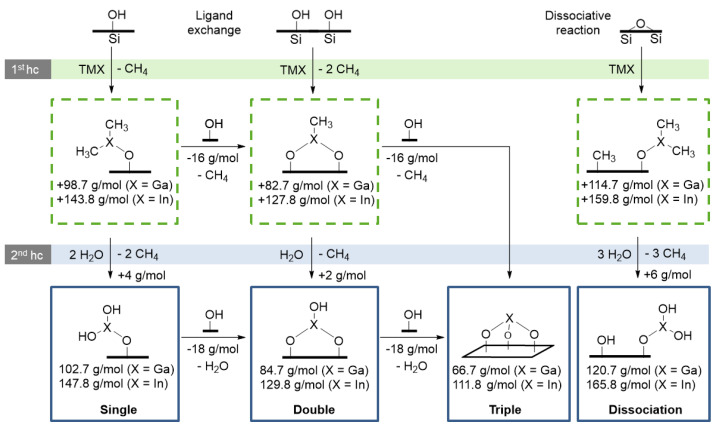
Possible reaction pathways during the ALD of trimethyl gallium or indium (TMG, TMI) and water on silica. Calculated mass changes per mol of used precursor are displayed as g/mol. Multiple dissociation steps do not lead to further mass change and are therefore excluded. The abbreviation *hc* implies an ALD half-cycle.

In the first cycle, the precursor TMX (X = Ga or In) can either react with Si-OH groups in a ligand exchange mechanism or with bridging oxygen of Si-O-Si (Figure 8). In the case of ligand exchange, the mass-uptake per mol of precursor depends on the number of ligands being replaced by silanol groups. The dissociative chemisorption of TMX on bridging oxygen results in the highest molar mass-uptake because all methyl groups are chemisorbed [93]. Subsequently, the methyl groups are exchanged by OH groups through a reaction with water. Thereby, methane is released as the only byproduct detected by the mass spectrometer.

Dividing the mass fraction of metal oxide (in-situ) by the moles of metal in the sample (ICP-OES) yields the average molar mass of chemisorbed precursor (in g/mol_Pre_). As a result, the GaO_x_ ALD process led to mass gains of 93.3 g/mol_TMG_ after the first, 95.4 g/mol_TMG_ after the second, and 94.9 g/mol_TMG_ after the third cycle. This indicates the single (+102.7 g/mol_TMG_) and double (+84.7 g/mol_TMG_) ligand exchange mechanism to occur as also estimated for AlO_x_ ALD [68]. However, the contribution of dissociative chemisorption cannot be neglected as methylated silica was found by NMR.

The number of surface OH-groups of silica was determined as 3 OH/nm^2^ via Grignard titration. Considering the specific surface area of GaO_x_/SiO_2_ and the mass fraction of gallium, the number of Ga atoms is calculated to be 3.75 Ga/nm^2^ after the first cycle. Therefore, dissociative chemisorption of TMG is necessary to reach the number of deposited gallium atoms. Any ratio of the ligand exchange reactions using the maximum number of 3 OH-groups/nm^2^ filled up with dissociative reactions to obtain 3.75 Ga/nm^2^ leads to around a 106 g/mol_TMG_ uptake. As the first cycle only led to 93.3 g/mol_TMG_, condensation of Ga(OH)_x_ might already happen within the first cycle, resulting in lower uptakes and M_2_O_3_ stoichiometry.

In the case of the InO_x_ ALD process, 151.8 g/mol_TMI_ is added in the first cycle. As the uptake is higher than the theoretical maximum by ligand exchange (147.8 g/mol_TMI_), dissociation clearly has a contribution. The mass fraction of indium suggests 4 In/nm^2^ being deposited in the first cycle. Any combination of ligand exchange reactions and dissociation, leading to 4 In/nm^2^ and consumption of 3 OH/nm2 (silica), would yield an uptake of 152.3 g/mol_TMI_. This value is close to the observed mass gain of 151.8 g/mol_TMI_ which proves the dissociative reaction occurs.

In the second and third cycles, the average molar mass of the chemisorbed precursor is 135.9 g/mol_TMI_ and 133.2 g/mol_TMI_ which are between a single (+147.8 g/mol_TMI_) and double (+129.8 g/mol_TMI_) ligand exchange mechanism (Figure 8). Therefore, chemisorption of TMI on In(OH)_x_ might favor the ligand exchange mechanism, whereas dissociative chemisorption has a higher contribution when reacting with SiO_2_. This phenomenon is accompanied by increased OH-group densities of 5–6 OH/nm^2^ after InO_x_ deposition, which allows more ligand exchange reactions. Moreover, less uptake per mole of used precursor might also be the result of condensation reactions between In-OH after the first cycle. Condensation leads to more intra-molecular In-O-In bonds which result in M_2_O_3_ character in higher cycles as suggested by XPS.

### 3.6. Estimated ALD Oxide Layer Thickness

The layers’ thickness is of special interest as it is comparable to literature values for flat substrates. It also serves as an approximate indicator for the formation of a closed monolayer as previously reported for AlO_x_ on powder [68]. Within the first cycles, the grown oxide follows the nature of the substrate’s surface, enforcing an amorphous structure with less density than the crystalline bulk oxide [103]. With further cycles, the layer eventually transforms into more crystalline material, yet the structure can only be estimated roughly.

In order to estimate the thickness of the InO_x_ layers, a density of 6.75 g/cm^3^ is assumed, which was determined by Chung et al. for amorphous, ALD-grown In_2_O_3_ [104]. Considering the respective mass fraction and surface area, InO_x_ grows 1.5 Å per cycle on average (gpc), generating a 4.6 Å thick oxide layer after three cycles (Table 4). The gpc is in the upper range of values reported for flat substrates which vary from 0.3 Å to around 2 Å [48,51,52,53]. Moreover, the growth increases with cycle number, which might be due to the favored formation of In(OH)_x_ in the first cycle [104].

For the amorphous GaO_x_ film, a reduced density of 5.5 g/cm^3^ is considered, calculated by Elam et al. [35]. As a result, the estimated gpc is 0.7 Å, leading to a layer thickness of 2.2 Å after the third cycle (Table 4). This is in agreement with values reported for flat substrates which are in the range of 0.5–1.5 Å [35,42]. As a comparison, TMA/H_2_O on the silica powder led to similar gpc of around 0.8 Å [68].

In cubic In_2_O_3_, the indium atoms are octahedrally coordinated with two different O-O distances depending on the miller plane orientation. Along the (110) plane, the average oxygen layer distance is approximately 2.5 Å and along the (111) plane, In_2_O_3_ grows less dense with a distance of 3.5 Å [105,106]. The calculated thickness for the second cycle (2.8 Å) indicates a defined monolayer being formed after the second and latest within the third cycle (4.6 Å). In β-Ga_2_O_3_, the gallium atoms are either octahedral or tetrahedral coordinated by oxygen with average distances of 2.8 and 3.0 Å [107,108]. Therefore, a monolayer is not considered to be formed within three cycles, as the estimated layer thickness reaches 2.2 Å after three cycles of GaO_x_ ALD. This is in line with findings for the AlO_x_ ALD on silica powder, as we estimated a monolayer to be formed around the third cycle [68].

## 4. Conclusions

The ALD processes for the deposition of gallium and indium oxide on mesoporous silica powder using trimethylgallium or trimethylindium and water were investigated. In-situ thermogravimetry confirmed self-limitation of the precursor-chemisorption and water was shown to be effective for the ligand removal. CHN analysis revealed carbon amounts below 0.2 wt% after InO_x_ ALD and the deposited carbon species in the case of GaO_x_ ALD were determined as methylated Si and Ga by FTIR and SS-NMR. Both processes showed distinct growth in every cycle, leading to mass fractions of 20–35 wt% GaO_x_ and 28–56 wt% InO_x_ within three ALD cycles. Thermogravimetric and ICP-OES data suggested a stoichiometry of Ga_2_O_3_ being present already after the first cycle. In the case of InO_x_ ALD, the transition from In(OH)_x_ to In_2_O_3_ with increasing cycle number was observed by XPS and confirmed by calculations based on thermogravimetric data and ICP-OES.

Despite their high mass fractions, the oxides were found to be highly dispersed and amorphous by STEM EDX-mappings and XRD. Additionally, the ALD processes led to specific surface areas of 260–340 m^2^/g for GaO_x_ and 140–280 m^2^/g for InO_x_ determined by N_2_ physisorption analysis. The layer thicknesses were estimated based on thermogravimetric data which revealed a gpc of 0.7 Å for GaO_x_ and 1.5 Å for InO_x_. In conclusion, the study provides new insights into the ALD of GaO_x_ and InO_x_ on mesoporous supports with high surface area. Both processes can potentially be applied for catalyst synthesis while the oxide loading is tunable by the cycle number. Supported ALD catalysts might be further investigated for application in hydrogenation and dehydrogenation catalysis.

## Figures and Tables

**Table 1:** Mass uptakes, molar uptakes, and total mass fractions of AlO_x_, GaO_x_, and InO_x_ on SiO_2_ during three cycles of ALD using TMX (X = A, G, I) and H_2_O at 150 °C (GaO_x_, InO_x_) or 200 °C (AlO_x_). Mass-uptake = ∆m/m_0_; molar-uptake = est.-Mol(M_2_O_3_)/m_0_; mass-fraction (frac.) = ∆m/(m_0_ + ∆m).

	AlO_x_/SiO_2_ [68]			GaO_x_/SiO_2_			InO_x_/SiO_2_		
ALD Cycles	Mass Up./%	Molar Up./mmol·g^−1^	Mass Frac./%	Mass Up./%	Molar Up. /mmol·g^−1^	Mass Frac./%	Mass Up./%	Molar Up. /mmol·g^−1^	Mass Frac./%
1	+11.9	+1.0	10.6	+24.3	+1.0	19.6	+38.7	+1.0	27.9
2	+11.4	+1.0	18.9	+16.3	+0.8	28.9	+44.3	+1.1	45.4
3	+13.4	+1.2	26.9	+14.2	+0.7	35.4	+45.8	+1.1	56.3
Sum	+36.7	+3.2	26.9	+54.8	+2.5	35.4	+128.8	+3.2	56.3

**Table 2:** Mass fractions of GaO_x_ and InO_x_ on SiO_2_ within three cycles of ALD using TMX (X = G, I) and water at 150 °C. Values are calculated from thermogravimetric data (MSB) and compared to ICP-OES data. Mass-fraction (frac.) = ∆m/(m_0_ + ∆m).

Sample	Mass Frac./%	^1^ M_2_O_3_ Frac./%	^2^ M(OH)_2_ Frac./%	Mass Frac./% (ICP-OES)
1c GaO_x_	19.6 (GaO_x_)	14.5 (Ga)	13.1 (Ga)	14.6 (Ga)
3c GaO_x_	35.4 (GaO_x_)	26.3 (Ga)	23.8 (Ga)	26.0 (Ga)
1c InO_x_	27.9 (InO_x_)	23.1 (In)	21.5 (In)	21.1 (In)
3c InO_x_	56.3 (InO_x_)	46.6 (In)	43.4 (In)	48.5 (In)

^1^ calculated from in-situ mass-fraction, assuming M_2_O_3_ species being deposited. ^2^ assuming M(OH)_2_ species being deposited on a single OH-group each.

**Table 3:** Specific surface areas (SA, calculated via BET) and total pore volume (PV) after ALD on SiO_2_ using TMX (X = A, G, I) and water at 200 °C (AlO_x_) or 150 °C (GaO_x_, InO_x_).

	AlO_x_/SiO_2_ [68]		GaO_x_/SiO_2_		InO_x_/SiO_2_	
ALD Cycles	SA/m^2^g^−1^	PV/cm^3^g^−1^	SA/m^2^g^−1^	PV/cm^3^g^−1^	SA/m^2^g^−1^	PV/cm^3^g^−1^
0	505	0.79	505	0.79	505	0.79
1	435	0.66	336	0.57	277	0.55
2	383	0.55	296	0.46	216	0.34
3	337	0.47	259	0.39	142	0.23

**Table 4:** Calculated layer thicknesses of the respective metal oxide on mesoporous silica after 1–3 cycles ALD using TMX (X = A, G, I) and water at 150 °C (gpc = average growth per cycle).

Sample [68]	Thickness/Å	Sample	Thickness/Å	Sample	Thickness/Å
1c AlO_x_	0.8	1c GaO_x_	0.9	1c InO_x_	1.1
2c AlO_x_	1.6	2c GaO_x_	1.6	2c InO_x_	2.8
3c AlO_x_	2.5	3c GaO_x_	2.2	3c InO_x_	4.6
gpc	0.8	gpc	0.7	gpc	1.5

## Data Availability

Data can be requested individually from the corresponding author.

## Supplementary Materials

The following supporting information can be downloaded at: https://www.mdpi.com/article/10.3390/nano12091458/s1, Figure S1: In-situ gravimetric monitoring of 4 ALD cycles GaO_x_ on SiO_2_ powder, Figure S2: Differential pore size distributions of GaO_x_ ALD modified SiO_2_, Figure S3: Differential pore size distributions of InO_x_ ALD modified SiO_2_, Figure S4: STEM-HAADF image and EDX-mappings of (a) 1 ALD cycle GaO_x_ on mesoporous SiO_2_, Figure S5: STEM-HAADF image and EDX-mappings of (b) 3 ALD cycle GaO_x_ on mesoporous SiO_2_, Figure S6: STEM-HAADF image and EDX-mappings of (c) 1 ALD cycle InO_x_ on mesoporous SiO_2_, Figure S7: STEM-HAADF image and EDX-mappings of (d) 3 ALD cycle InO_x_ on mesoporous SiO_2_, Figure S8: STEM-HAADF image and EDX-mappings of (e) impregnated In_2_O_3_ (22 wt% In) on mesoporous SiO_2_, Table S1: Metal-uptakes of Al, Ga and In on SiO_2_ within 3 cycles of ALD using TMX (X = A, G, I) and water, Table S2: Estimated (blue) specific surface areas (ESA) and total pore volumes (EPV) of ALD-modified SiO_2_ based on model 1, Table S3: Estimated (blue) specific surface area (ESA) and mass-uptake (EUp) of ALD-modified SiO_2_ based on model 2, Table S4: Estimated (blue) surface area (ESA) and pore volume (EPV) of ALD-modified SiO_2_ based on model 3, Table S5: Fit parameters for the XPS scans of the Ga3d, In3d and O1s regions after GaO_x_ and InO_x_ ALD on SiO_2_, Scheme S1: Schematic description of model 1, based on two ALD cycles of InO_x_ on SiO_2_, Scheme S2: Schematic description of model 2 (Core-shell), based on two ALD cycles of InO_x_ on SiO_2_, Scheme S3: Schematic description of model 3 (volumetric), based on two ALD cycles of InO_x_ on SiO_2_.
