# Supplementary material for: Synthesis of High Surface Area—Group 13—Metal Oxides via Atomic Layer Deposition on Mesoporous Silica
Source: Nanomaterials (Basel). 2022 Apr 25;12(9):1458. doi: 10.3390/nano12091458 (PMC9104076; doi:10.3390/nano12091458)
Supplement: Supplementary file 1 [file nanomaterials-12-01458-s001.zip › nanomaterials-1680430-supplementary.pdf]

nanomaterials

MDPI

Supplementary Material

Synthesis of High Surface Area—Group 13—Metal Oxides via Atomic Layer Deposition on Mesoporous Silica

Robert Baumgarten [1], Piyush Ingale [1], Kristian Knemeyer [1], Raoul Naumann d'Alnoncourt [1,*], Matthias Driess [1,2] and Frank Rosowski [1,3]

[1] BasCat—UniCat BASF JointLab, Technische Universität Berlin, Hardenberstraße 36, 10623 Berlin, Germany; r.baumgarten@bascat.tu-berlin.de (R.B.); p.ingale@bascat.tu-berlin.de (P.I.); k.knemeyer@bascat.tu-berlin.de (K.K.); matthias.driess@tu-berlin.de (M.D.); frank.rosowski@basf.com (F.R.)

[2] Institut für Chemie: Metallorganik und Anorganische Materialien, Technische Universität Berlin, Straße des 17. Juni 135, 10623 Berlin, Germany

[3] Process Research and Chemical Engineering, BASF SE, Carl-Bosch-Straße 38, 67056 Ludwigshafen, Germany

* Correspondence: r.naumann@bascat.tu-berlin.de; Tel.: +49-30-314-73683

1. Synthesis of Reference Samples

Materials: Silica powder (SiO_2 amorphous, ≥99%, high-purity grade (Davisil Grade 636), average pore size 60 Å, 35–60 mesh particle size, specific surface area 505 m^2/g, Sigma-Aldrich). Gallium and indium nitrate hydrate ($Ga(NO_3)_3 \cdot xH_2O$ and $In(NO_3)_3 \cdot xH_2O$, 99.9% trace metals basis, Merck, Germany). Water (H_2O, CHROMASOLV®, for HPLC, Riedel-de Haën/ Honeywell Specialty Chemicals Seelze GmbH, Seelze, Germany).

Protocol: Reference samples, supported on SiO_2, were synthesized via incipient wetness impregnation (IWI). For the synthesis of Ga_2O_3/SiO_2 and In_2O_3/SiO_2, the respective nitrate hydrate was added to HPLC-grade water to obtain the required volume for IWI of one gram of silica. After impregnation, the samples were dried in air at 80 °C for 12 h. Subsequently, the samples were calcined at 500°C for 3 h under 20% O_2/N_2 (syn-air). The three-cycle ALD samples were calcined under same conditions for comparison.

Equations

$$\text{Added mass/mol(precursor)} = \frac{\text{mass(oxide)} \times \text{M(metal)}}{\text{mass(metal)}} \tag{1}$$

Consideration: Amount of deposited metal equals chemisorbed precursor

$$\text{Layer thickness(oxide)} = \frac{\text{mass(oxide)}}{\rho\text{(oxide)} \times \text{surface area(substrate)}} \tag{2}$$

Consideration: Flat substrate surface and even oxide layer

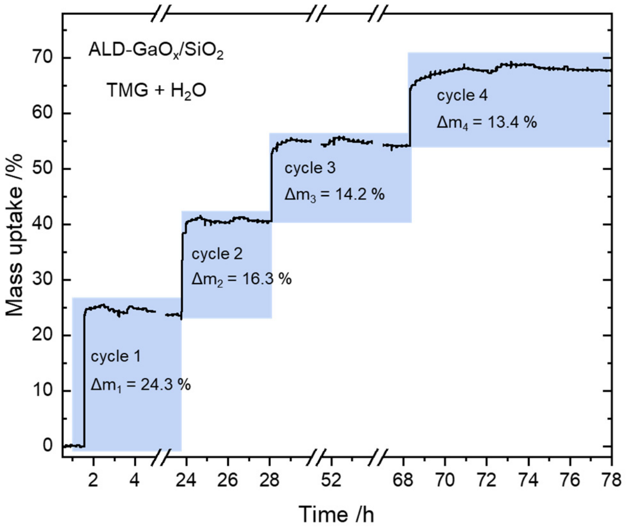

Figure S1. *In-situ* gravimetric monitoring of 4 ALD cycles GaO_x on SiO_2 powder using the process of TMG/H_2O at 150 °C. Mass-uptake = $\Delta m/m_0$ (m_0 = SiO_2).

Table S1. Metal-uptakes of Al, Ga and In on SiO_2 within 3 cycles of ALD using TMX (X = A, G, I) and water. Values are calculated from thermogravimetric data (MSB) as described in chapter 3.1. Mass-uptake = $\Delta m/m_0$ (m_0 = SiO_2).

Cycle Nr.	Al-Metal [1] Uptake /%	Ga-Metal Uptake /%	In-Metal Uptake /%	Uptake Ratio (Al : Ga : In)	Molar Mass Ratio (Al:Ga:In)
1 cycle	+ 8.7	+ 18.1	+ 29.9	1:2.1:3.4	1:2.6:4.3 (native)
Ø 3 cycles	+ 7.5	+ 13.6	+ 35.5	1:1.8:4.7	

2. Basic Consideration and Rationalization of the Changes in Surface Area and Pore Volume

2.1. Approximation (Density):

The specific surface areas are estimated based on the ALD-induced change of the substrate-mass, assuming no change of the exposed surface area (as discussed in chapter 3.2). The estimated (specific) surface areas and pore volumes are displayed in Table S2 (blue).

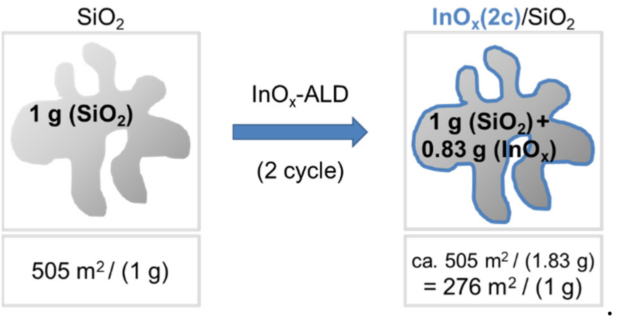

Scheme S1. Schematic description of model 1, based on two ALD cycles of InO_x on SiO_2.

Table S2. Estimated (blue) specific surface areas (ESA) and total pore volumes (EPV) of ALD-modified SiO_2 based on model 1. Values are compared to the measured values (green) from N_2 physisorption analysis (SA, PV). Mass-uptakes derive from thermogravimetric data. ESA = SA(SiO_2)/(1+mass-uptake), EPV = PV(SiO_2)/(1+mass-uptake).

	GaO_x/SiO_2				InO_x/SiO_2			
ALD cycle	ESA /m^2 g^{-1}	SA /m^2 g^{-1}	EPV /cm^3 g^{-1}	PV /$cm^3 g^{-1}$	ESA /m^2 g^{-1}	SA /m^2 g^{-1}	EPV /cm^3 g^{-1}	PV /cm^3 g^{-1}
0 (SiO_2)	505		0.79		505		0.79	
1	406	336	0.64	0.57	364	277	0.57	0.55
2	359	296	0.56	0.46	276	216	0.43	0.34
3	326	259	0.51	0.39	221	142	0.35	0.23

2.2. Approximation (Geometric, Core-Shell):

The total surface area of silica is concentrated on (non-porous) spheres with a diameter of 4.48 nm (best fit), density of 2.65 g/cm^3 and no inter-particle volume. The sphere diameters are increased by a thin ALD layer of higher density (5.5 g/cm^3 for GaO_x and 6.75 g/cm^3 for InO_x, ref.: chapter 3.6). The layer is homogeneously distributed and its thickness is calculated using equation (2) (see chapter 3.6). The non-mass-related surface area of the sphere increases slightly while the mass-related (specific) surface area decreases drastically (Table S3).

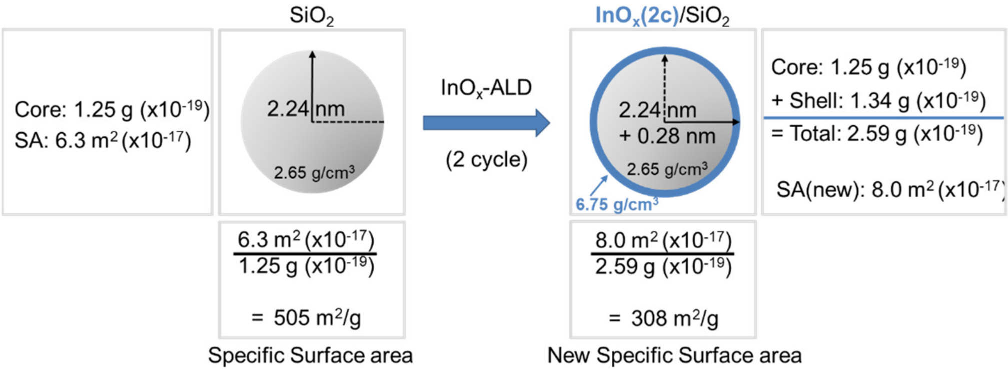

Scheme S2. Schematic description of model 2 (Core-shell), based on two ALD cycles of InO_x on SiO_2.

Table S3. Estimated (blue) specific surface area (ESA) and mass-uptake (EUp) of ALD-modified SiO_2 based on model 2. Values are compared to the measured values (green) from N_2 physisorption analysis (SA). Mass-uptakes (Up) derive from thermogravimetric data. ESA = SA(new)/(1 + mass-uptake), EUp = $\Delta m/m_0$ ($m_0 = SiO_2$).

	GaO_x/SiO_2				InO_x/SiO_2			
ALD cycle	ESA /m^2 g^{-1}	SA /m^2 g^{-1}	EUp /wt%	Up /wt%	ESA /m^2 g^{-1}	SA /m^2 g^{-1}	EUp /wt%	Up /wt%
0 (SiO_2)	505		0		505		0	
1	434	336	26	24	399	277	39	39
2	393	296	40	48	308	216	108	83
3	364	259	67	55	252	142	191	129

2.3. Approximation (Volumetric):

The total pore volume and surface area of silica is concentrated inside spherical pores with a diameter of 9.39 nm (best fit) without connections between the pores. The number of pores is normalized to 1.82×10^{18} to reach the total pore volume and surface area of 1 g SiO_2 (0.79 cm^3 and 505 m^2). The pore diameters are decreased by a homogeneously

distributed, thin ALD layers (see chapter 3.6 or equation (2)). Consequently, the inner surface area and pore volume of each pore decreases and the mass-based (specific) values as well (Table S4).

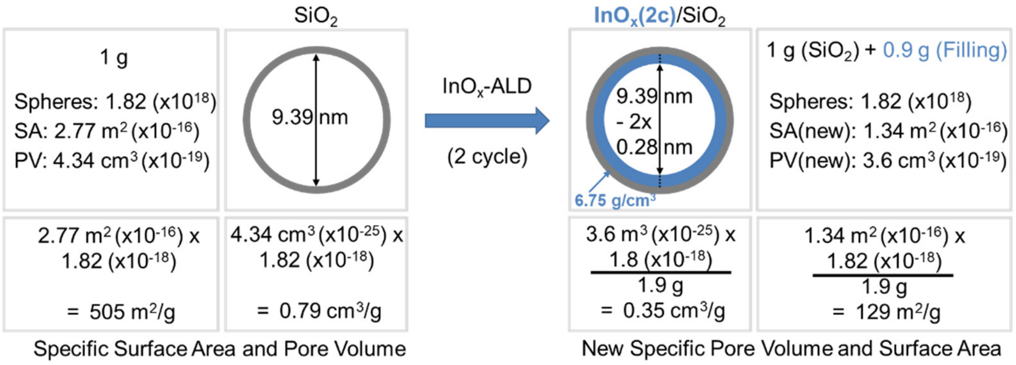

Scheme S3. Schematic description of model 3 (volumetric), based on two ALD cycles of InO_x on SiO_2.

Table S4. Estimated (blue) surface area (ESA) and pore volume (EPV) of ALD-modified SiO_2 based on model 3. Values are compared to the measured values (green) from N_2 physisorption analysis (SA).

	GaO_x/SiO_2						InO_x/SiO_2					
**ALD cycle**	**ESA /m² g⁻¹**	**SA /m² g⁻¹**	**EPV /cm³**	**PV /cm³**	**EUp /wt%**	**Up /wt%**	**ESA /m² g⁻¹**	**SA /m² g⁻¹**	**EPV /cm³g⁻¹**	**PV /cm³g⁻¹**	**EUp /wt%**	**Up /wt%**
0 (SiO_2)	505		0.79		0	0	505		0.79		0	0
1	214	336	0.60	0.57	25	24	193	277	0.54	0.55	37	39
2	181	296	0.50	0.46	43	48	129	216	0.35	0.34	90	83
3	159	259	0.43	0.39	58	55	93	142	0.24	0.23	142	129

Conclusion: The three shown models are only rough approximations and do not fully reflect the reality. An exact model would lie somewhere in-between, yet with unknown proportions. However, the mass-uptakes, changes in specific surface areas and pore volumes are mostly in line with what was observed *via* measurements. Finally, the models justify and rationalize the drastically appearing decrease in surface areas and pore volumes by metal oxide ALD.

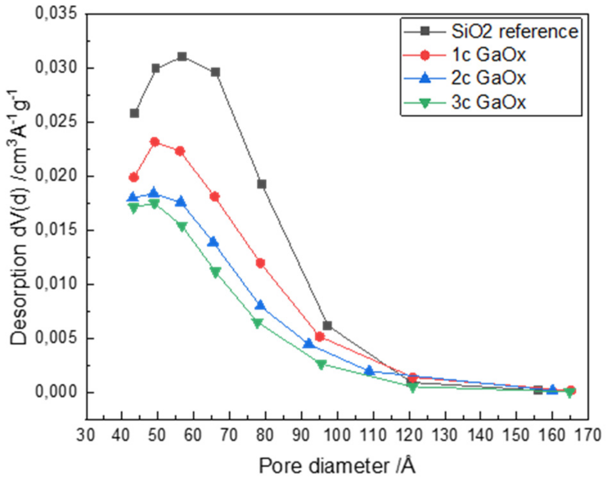

Figure S2. Differential pore size distributions of GaO_x ALD modified SiO_2, determined by the N_2 desorption branches and application of the BJH method. 1-3 cycles of GaO_x ALD were conducted using TMG and water at 150°C.

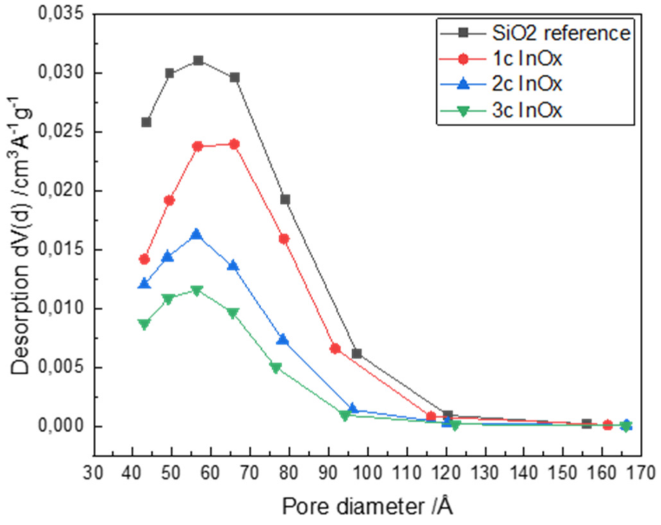

Figure S3. Differential pore size distributions of InO_x ALD modified SiO_2, determined by the N_2 desorption bracnhes and application of the BJH method. 1-3 cycles of InO_x ALD were conducted using TMI and water at 150°C.

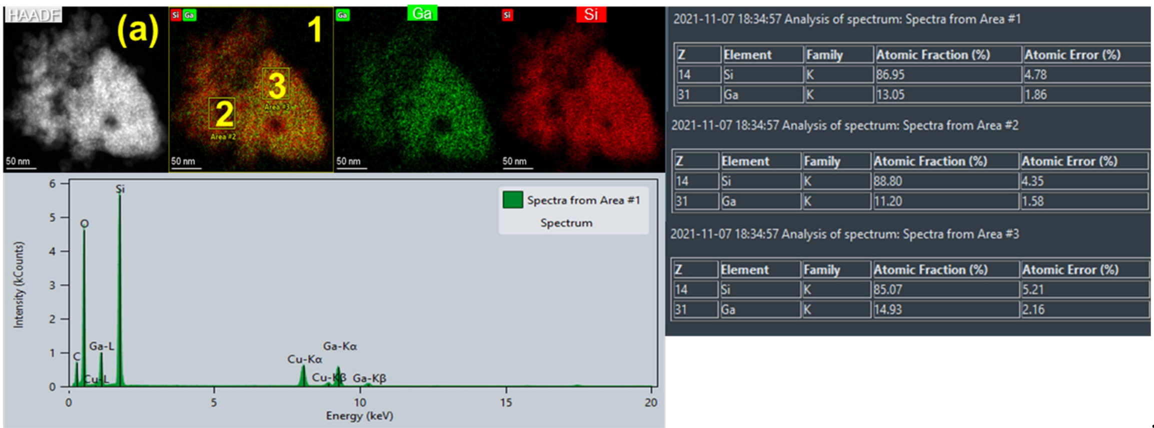

2021-11-07 18:34:57 Analysis of spectrum: Spectra from Area #1

Z	Element	Family	Atomic Fraction (%)	Atomic Error (%)
14	Si	K	86.95	4.78
31	Ga	K	13.05	1.86

2021-11-07 18:34:57 Analysis of spectrum: Spectra from Area #2

Z	Element	Family	Atomic Fraction (%)	Atomic Error (%)
14	Si	K	88.80	4.35
31	Ga	K	11.20	1.58

2021-11-07 18:34:57 Analysis of spectrum: Spectra from Area #3

Z	Element	Family	Atomic Fraction (%)	Atomic Error (%)
14	Si	K	85.07	5.21
31	Ga	K	14.93	2.16

Figure S4. STEM-HAADF image and EDX-mappings of (**a**) 1 ALD cycle GaO_x on mesoporous SiO_2. For analysis of Area #1 the whole image was selected.

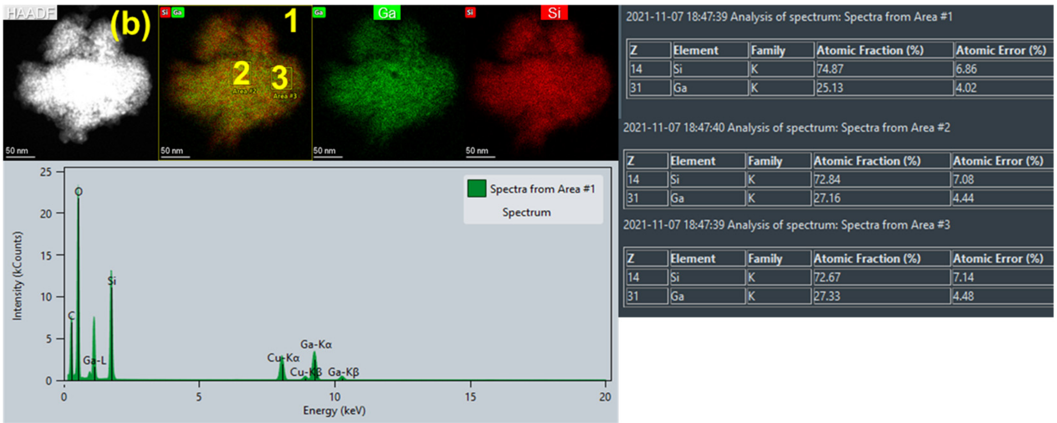

2021-11-07 18:47:39 Analysis of spectrum: Spectra from Area #1

Z	Element	Family	Atomic Fraction (%)	Atomic Error (%)
14	Si	K	74.87	6.86
31	Ga	K	25.13	4.02

2021-11-07 18:47:40 Analysis of spectrum: Spectra from Area #2

Z	Element	Family	Atomic Fraction (%)	Atomic Error (%)
14	Si	K	72.84	7.08
31	Ga	K	27.16	4.44

2021-11-07 18:47:39 Analysis of spectrum: Spectra from Area #3

Z	Element	Family	Atomic Fraction (%)	Atomic Error (%)
14	Si	K	72.67	7.14
31	Ga	K	27.33	4.48

Figure S5. STEM-HAADF image and EDX-mappings of (**b**) 3 ALD cycle GaO_x on mesoporous SiO_2. For analysis of Area #1 the whole image was selected.

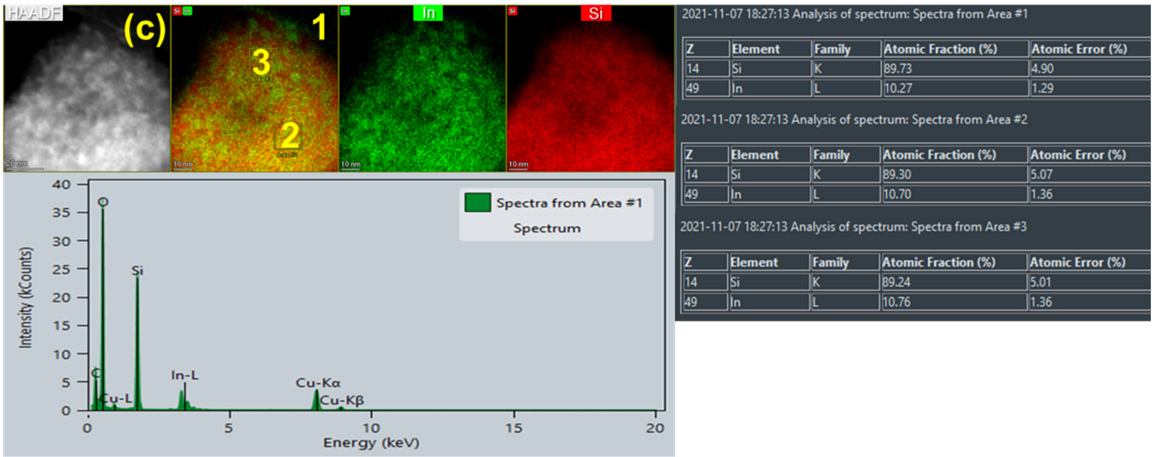

2021-11-07 18:27:13 Analysis of spectrum: Spectra from Area #1

Z	Element	Family	Atomic Fraction (%)	Atomic Error (%)
14	Si	K	89.73	4.90
49	In	L	10.27	1.29

2021-11-07 18:27:13 Analysis of spectrum: Spectra from Area #2

Z	Element	Family	Atomic Fraction (%)	Atomic Error (%)
14	Si	K	89.30	5.07
49	In	L	10.70	1.36

2021-11-07 18:27:13 Analysis of spectrum: Spectra from Area #3

Z	Element	Family	Atomic Fraction (%)	Atomic Error (%)
14	Si	K	89.24	5.01
49	In	L	10.76	1.36

Figure S6. STEM-HAADF image and EDX-mappings of (**c**) 1 ALD cycle InO_x on mesoporous SiO_2. For analysis of Area #1 the whole image was selected.

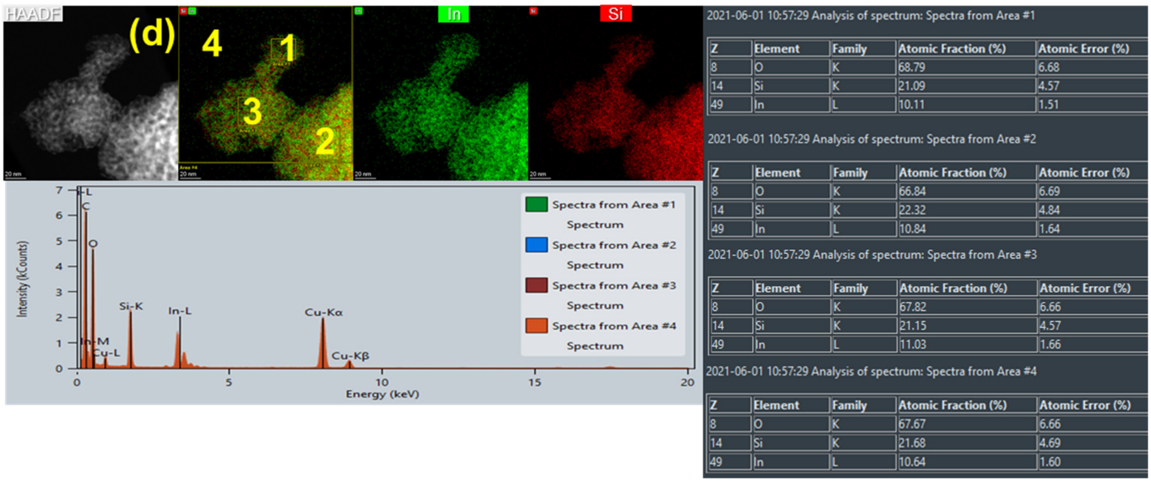

Figure S7. STEM-HAADF image and EDX-mappings of (**d**) 3 ALD cycle InO_x on mesoporous SiO_2. For analysis of Area #4 the whole image was selected.

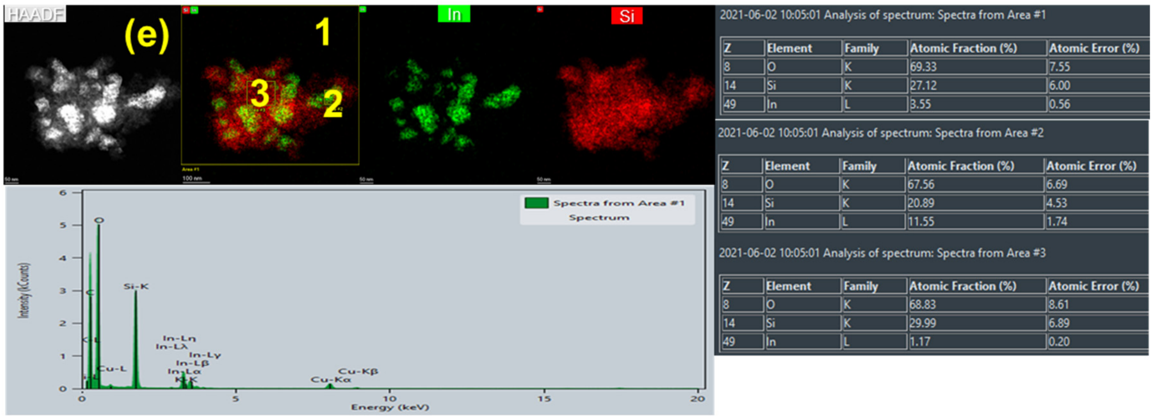

Figure S8. STEM-HAADF image and EDX-mappings of (**e**) impregnated In_2O_3 (22 wt% In) on mesoporous SiO_2. For analysis of Area #1 the whole image was selected.

Table S5. Fit parameters for the XPS scans of the Ga3d, In3d and O1s regions after GaO_x and InO_x ALD on SiO_2. Shown are the peak positions, full width at half maxima (FWHM), the used L/G Mix and area ratios.

	**Ga3d region (after GaO_x ALD)**						
	O2s			Ga3d (Ga_2O_3)			
Sample	Peak BE (eV)	FWHM (eV)	L/G Mix (%)	Peak BE (eV)	FWHM (eV)	L/G Mix (%)	Area ratio
GaO_x/SiO_2 (1 cycle)	24.68	3.23	30	20.66	2.78	30	0.62 to 1
GaO_x/SiO_2 (3 cycle)	24.87	3.45	30	20.80	2.72	30	0.30 to 1

	**In3d region (after InO_x ALD)**						
	$In3d_{3/2}$			$In3d_{5/2}$			
Sample	Peak BE (eV)	FWHM (eV)	L/G Mix (%)	Peak BE (eV)	FWHM (eV)	L/G Mix (%)	Area ratio
InO_x/SiO_2 (1 cycle)	452.64	2.38	30	445.10	2.38	30	0.69 to 1
InO_x/SiO_2 (2 cycle)	452.47	1.89	30	444.93	1.89	30	0.69 to 1
InO_x/SiO_2	452.32	1.85	30	444.78	1.85	30	0.69 to 1

(3 cycle)

	O1s region (after InO_x ALD)						
	O-Si (SiO_2)			O-In (In_2O_3)			
Sample	Peak BE (eV)	FWHM (eV)	L/G Mix (%)	Peak BE (eV)	FWHM (eV)	L/G Mix (%)	Area ratio
InO_x/SiO_2 (1 cycle)	532.25	2.44	30	530.00	2.44	30	1 to 0.05
InO_x/SiO_2 (3 cycle)	532.15	1.91	30	530.22	1.90	30	1 to 0.30

References

1. Strempel, V.E.; Knemeyer, K.; Naumann d'Alnoncourt, R.; Driess, M.; Rosowski, F. Investigating the Trimethylaluminium/Water ALD Process on Mesoporous Silica by In Situ Gravimetric Monitoring. Nanomaterials 2018, 8, 365. https://doi.org/10.3390/nano8060365.
